# Self-esteem and professional identity among male nurses and male nursing students: mediating roles of perceived prejudice and psychological distress

**DOI:** 10.3389/fpsyg.2023.1176970

**Published:** 2023-06-13

**Authors:** Xiaoqin Wu, Xu You, Jinyuan Pu, Junping Li, Wenzhi Wu, Xiao Ma, Qing Long, Yunqiao Zhang, Xinling Zhao, Zeyi Guo, Xiang Cao, Fangjun Tu, Yong Zeng

**Affiliations:** ^1^Department of Psychiatry, The Second Affiliated Hospital of Kunming Medical University, Kunming, China; ^2^Department of Psychiatry, Honghe Second People’s Hospital, Honghe, China

**Keywords:** male nursing students, male nurses, perceived prejudice, psychological distress, professional identity, self-esteem

## Abstract

**Introduction:**

There are not enough nurses around the world, and there are even fewer male nurses. It has not been easy for men to become nurses because of stereotypes about the roles of men and women in the workplace, which lead to prejudice and discrimination. This study explored how the self-esteem of male nurses and male nursing students affects their professional identity in an environment where stereotypes and social prejudice exist. This study also examined the differences of relevant variables in different sociodemographic characteristics of the research subjects in a Chinese social context.

**Methods:**

By purposive and snowball sampling, 464 male nurses and male nursing students were surveyed through questionnaires from November 2021 to January 2022. Data analysis was performed using SPSS 25.0 and PROCESS Macro 3.3.

**Results:**

Self-esteem could indirectly affect professional identity through perceived prejudice and psychological distress. Nonetheless, self-esteem still had a significant direct effect on professional identity. The total mediating effect accounted for 32.816% of the total effect, and the direct effect accounted for 67.184% of the total effect. Also of note was that 81.7% of participants reported experiencing psychological distress.

**Discussion:**

To improve the professional identity of male nurses and male nursing students, nursing educators and administrators should do the following: protect and improve their self-esteem; take steps to reduce social prejudice against them; value their mental health and alleviate their psychological distress.

## 1. Introduction

The history of male nursing can be traced back to the infancy of the nursing profession. Men had already played an important functional role as nurses in military and nonmilitary activities (disease and plague outbreaks) from the 4th and 5th centuries and continued to be the leading providers of health care services into the 16th century (Arif and Khokhar, 2017). Until the mid-19th century, when Nightingale pioneered modern nursing, she firmly believed that nursing was a job for women, and male nurses were gradually marginalized (Mackintosh, 1997). After that, women dominated the nursing profession, and it became more difficult for men to become nurses, influenced by gender role stereotypes (Egeland and Brown, 1988). Gender role stereotypes are overt societal beliefs about the functional characteristics of men and women that inevitably influence career choices and development (Kagan, 1964). As a result, men in nursing are often perceived as violating masculine norms or deviating from male gender roles and are thus labeled in negative ways, such as incapable, troublemakers, effeminate, homosexual, abnormal, and strange (Harding, 2007; Moss-Racusin et al., 2010; Adeyemi-Adelanwa et al., 2016; Stanley et al., 2016). Stereotypes can be a source of social prejudice and discrimination against male nursing students and male nurses (Nelson and Belcher, 2006; Crandall et al., 2011). Male nurses often experience being denied care by female patients; also, some male nurses reported that they are easily watched in the hospital, making them uncomfortable (Chang and Jeong, 2021). Male nursing students are prone to experiencing ridicule, isolation, and loneliness, and negative attitudes toward male nursing students are evident, especially among male non-nursing students (MacWilliams et al., 2013; Clow et al., 2014). In addition, male nursing students often feel isolated, excluded, and treated differently in academic and clinical settings; for example, they are often singled out by female classmates or faculty for patient roles, and they learn to remain silent rather than actively and enthusiastically speak up in a predominantly female group learning environment (Stott, 2007). There is no denying the persistence of social stereotypes, prejudice, and discrimination against male nursing students and male nurses that may drive them away from the nursing profession (Lou et al., 2007; Kox et al., 2022).

Prejudice is a negative evaluation of a social group or person based primarily on the individual’s group membership (Crandall and Eshleman, 2003). Perceived prejudice is an individual’s perception that negative external evaluations of him or herself do not correspond to reality but are due to group membership (Yao and Yang, 2017). Prejudice is still at the level of negative attitudes. Discrimination includes negative attitudes and rises to adverse treatment such as rejection and avoidance; prejudice may be a better predictor of discrimination than stereotypes (Thornicroft et al., 2007). Many qualitative studies have found male nursing students and male nurses to perceive social prejudice against them (Gao et al., 2019; Saleh et al., 2020; Lyu et al., 2022; Subu et al., 2022), but more quantitative research is needed (Feng et al., 2016, 2019). In China, men studying nursing or working in nursing are looked down upon due to the low social status of nurses and the influence of the traditional Chinese culture of male preference and men’s superiority to women. As a result, male nursing students and male nurses in China may suffer from more social prejudice. Prejudice and discrimination against a group are detrimental to the physical and mental health of members of this group, which may increase their psychological distress (Stuber et al., 2008). Psychological distress refers to non-specific mental health problems such as anxiety and depression (Liu, 2016). Nursing students and nurses are usually under high stress, which is closely related to psychological distress (Watson et al., 2009; Smith and Yang, 2017; Foster et al., 2020), and the prevalence of psychological distress is generally not low, especially for nurses is high. The nurses’ workload in China is high; in most public hospitals, nurses work 40 h a week (Lin et al., 2009). The prevalence of psychological distress among Chinese nurses was 83.3%, with 34 male and 428 female nurses participating in this survey (Liu et al., 2021). The prevalence rate of female nurses in China was 85.5% (Zou et al., 2016). The prevalence of psychological distress among Chinese nursing students was 55.8%, with 375 male and 1,366 female nursing students participating in this survey (Qi et al., 2022). However, one study showed that the prevalence of psychological distress among male nursing students in China was 82.2% (Feng et al., 2019). Few studies focus on the mental health of male nursing students or male nurses like this. Since the number of males in the nursing student and nurse population is too small compared to females, more studies should be conducted on male nursing students and male nurses for their psychological distress to be more clearly presented. Overall, male nursing students and male nurses are a minority compared to their female counterparts and are in a particular environment where stereotypes, prejudice, and discrimination exist. Their mental health needs more attention.

The global shortage of nurses is receiving increasing attention (Marć et al., 2019). However, in the global shortage of nurses, the shortage of male nurses is more prominent than that of female nurses. Between 2017 and 2019, the proportion of registered male nurses was 11.1% in Australia, 10.7% in the United Kingdom, 9.1% in the United States, and only 2% in China (Younas et al., 2022). Attracting more men into nursing and reducing their attrition would help alleviate the nursing shortage (Brady and Sherrod, 2003), and gender diversity would help modern nursing evolve (Sullivan, 2000). However, nursing schools get very few male students, and male nursing students are likelier to leave the nursing profession than female nursing students (McLaughlin et al., 2010; Moore et al., 2020). Moreover, male nurses have a lower professional identity than female nurses (Liu et al., 2022), and professional identity is essential in their intentions to leave the profession (Sabanciogullari and Dogan, 2015).

Professional identity is how nurses or nursing students see themselves as part of the nursing profession, how they feel about it, and what it means to society (Ohlén and Segesten, 1998). Nurses’ professional identity positively impacts both subjective well-being and job performance (Ren et al., 2021; Yu et al., 2022). In addition, nurses with a high occupational identity tend to have high job satisfaction, enhancing retention intentions (Hanum et al., 2023). However, nurses with low occupational identity and job satisfaction were likelier to leave (Sabanciogullari and Dogan, 2015). Nurses are often seen as subordinate to physicians, and nursing is perceived as low-skilled and bedside care (Teresa-Morales et al., 2022). For a long time, nurses could not shake the image of low social status, and low self-esteem and low professional identity often accompany nurses (Ten Hoeve et al., 2014). In addition, male nursing students and male nurses in China typically have a lower sense of professional identity than their female counterparts (Mao et al., 2021). Studies have shown that Chinese male nurses with a high sense of professional identity are willing to engage in more work and thus promote professional success (Wu et al., 2022). Nurses’ professional identity is a dynamic developmental process, which means that the professional identity of male nursing students and male nurses can be reconstructed or strengthened (Johnson et al., 2012; Van der Cingel and Brouwer, 2021). Studies have shown that nurses’ self-esteem is closely related to their professional identity (Ohlén and Segesten, 1998; Olthuis et al., 2007). Self-esteem is one’s attitude toward oneself, and it plays a crucial role in personality building, psychological balance, and environmental adaptation (Doré, 2017). Self-esteem has been widely studied in the behavioral and social sciences, and the benefits of high self-esteem have been affirmed time and time again (Orth and Robins, 2022). Tajfel and Turner proposed the social identity theory, in which they considered social identity to be the self-image that individuals perceive themselves to have in the group to which they belong, as well as the emotional and value experiences they have as members of the group (Tajfel and Turner, 1979). Based on social identity theory, we argue that male nursing students and male nurses may harm their self-esteem and social identity through social categorization and social comparison (images of nurses’ low social status and perceived prejudice of male nursing students and male nurses), resulting in low professional identity and eventual departure from the nursing profession. We suggest that self-esteem and professional identity may be vital for male nursing students and male nurses and that the relationship between the two needs to be further explored in a setting where stereotypes and social prejudices exist.

Additionally, this study also examined the differences of relevant variables in different sociodemographic characteristics of the research subjects. Graduates from high schools in China can apply for the university majors they wish to pursue. Still, if their grades do not meet the requirements, they might end up being placed in a major for which not many people apply. Usually, nursing is one of those majors that few people apply for. Studies have found that male nurses and male nursing students who applied for the nursing major as their first choice typically had a stronger sense of professional identity than those who did not (Zhang et al., 2018; Zhou et al., 2021). Another study has found that male nursing students at three-year colleges had a higher professional identity than junior male nurses (Chen et al., 2020). However, few studies still compare the professional identity of male nursing students and male nurses. For a long time before, a junior college or below educational level was sufficient to serve as a nurse in China (Jiang et al., 2002). Still, as the nursing profession has grown, hospitals have begun recruiting people with higher educational levels to be nurses (Yang et al., 2021). In summary, this study additionally addressed the following questions: (a) Is there a statistical difference between the professional identity of male nursing students and male nurses who applied for nursing as their first-choice major and that of those who did not?; (b) Is there a statistical difference between the professional identity of male nursing students and that of male nurses?; and (c) Is there a statistical difference in the professional identity of the research subjects at different levels of education?

### 1.1. The impact of self-esteem on professional identity

Self-esteem positively predicts professional identity; individuals with high levels of self-esteem tend to have a higher professional identity (Motallebzadeh and Kazemi, 2018). Among the many factors influencing nursing students’ professional identity, self-esteem and professional values are significant predictors (Min et al., 2021). Studies have shown that Chinese male nursing students’ self-esteem and professional identity are positively correlated (Cao et al., 2016). The professional identity of male nursing students and male nurses is subject to constant change, and affirming the importance and value of male nurses themselves can help to increase self-esteem and thus enhance professional identity (Zhang, 2019). There are far fewer male nursing students and male nurses in China than their female counterparts, and they are more willing to leave the profession (Tong et al., 2021). Therefore, improving the professional identity of male nursing students and male nurses is essential. Although there are few studies on self-esteem and professional identity, it is possible to consider self-esteem a critical and influential factor in improving the professional identity of male nursing students and male nurses.

Therefore, the present study proposes hypothesis 1: self-esteem of male nursing students and male nurses can directly and positively predict professional identity.

### 1.2. Mediating role of perceived prejudice

Perceived prejudice and perceived discrimination both focus on subjective feelings; individuals feel that they have suffered prejudice and discrimination. Previous studies have found that self-esteem negatively predicts perceived discrimination in other groups that are discriminated against (Ji et al., 2019; Maqsood et al., 2021). Feng et al. (2019) found that the self-esteem of Chinese male nursing students negatively predicted perceived prejudice; they also found that the more substantial the perceived prejudice of male nursing students, the lower their professional satisfaction would be and, ultimately, the less willing they would be to become a nurse (Feng et al., 2016). The less willing they are to become nurses, the less they may identify with the nursing profession. However, the job satisfaction of Chinese male nurses was significantly and positively correlated with professional identity (Zhu, 2019). In addition, studies have shown that some negative experiences and feelings in the clinical learning environment may harm the professional identity of Chinese nursing students (Wu et al., 2020). In China, some male nursing students were reluctant to admit their major was nursing in front of their new acquaintances (Chen et al., 2020). Some male nurses talk about the social prejudice they had experienced from their nursing student days to when they joined the workforce and how it has affected their professional identity (Zhang, 2019). However, there is a lack of quantitative research on the impact of perceived prejudice on professional identity among male nursing students and male nurses. Due to the traditional Chinese culture, there is a more significant societal prejudice against men studying and working in nursing. However, high self-esteem is associated with coping with stress, adaptive adjustment, well-being, success, and satisfaction (Mann et al., 2004). In summary, high self-esteem may allow male nursing students and male nurses to perceive less prejudice and thus maintain a higher professional identity.

Therefore, the present study proposes hypothesis 2: self-esteem negatively predicts perceived prejudice, and then perceived prejudice negatively predicts professional identity. That is, for male nurses and male nursing students, perceived prejudice is a mediating factor in the link between self-esteem and professional identity.

### 1.3. Mediating role of psychological distress

Self-esteem is one of the core elements of mental health and a key element in promoting mental health (Mann et al., 2004). The vulnerability model suggests low self-esteem can lead to depression (Beck, 1967), while the terror management theory suggests that self-esteem can act as a buffer for anxiety (Pyszczynski et al., 2004). Studies have shown that nurses’ self-esteem can negatively predict psychological distress (Feng et al., 2018; Duran et al., 2021; Liu et al., 2021). Feng et al. (2019) found that the self-esteem of Chinese male nursing students also negatively predicted psychological distress. In addition, the level of psychological well-being of college students has a positive impact on their professional identity (Strauser et al., 2008). In a study of student teachers, anxiety was negatively associated with career identity (Zhao et al., 2022). However, there is a lack of studies on the impact of psychological distress on occupational identity among male nursing students and male nurses. Low self-esteem is a risk factor for various mental disorders; high self-esteem is associated with mental health, well-being, success, and satisfaction (Mann et al., 2004). In summary, high self-esteem may promote the mental health of male nursing students and nurses, reduce their psychological distress, and thus maintain a high professional identity.

Therefore, the present study proposes hypothesis 3: self-esteem negatively predicts psychological distress, and then psychological distress negatively predicts professional identity. In other words, psychological distress mediates the link between self-esteem and professional identity among male nurses and male nursing students.

### 1.4. The chain mediating effect of perceived prejudice and psychological distress

Based on Lazarus and Folkman’s coping theory (Folkman et al., 1986), the perceived prejudice of male nursing students and male nurses is a stressor. However, it is currently impossible to eliminate social prejudice, which will likely trigger their adverse emotions and poor coping behaviors. In China, 72% of male nursing students believe that social perceptions of the nursing profession cause significant stress (Chen and Mei, 2014). Furthermore, perceived stress among male nurses in China negatively affects professional identity (Chen et al., 2022). Therefore, we suggest that more perceived prejudice among male nursing students and male nurses indicates higher stress, which may cause them psychological distress and low professional identity. Numerous studies have shown that perceived discrimination negatively affects a person’s physical and mental health through complex biopsychosocial interactions (Pascoe and Richman, 2009; Todorova et al., 2010; Lockwood et al., 2018). The more discrimination an individual perceives, the greater the risk of psychological distress (Wamala et al., 2007; Schmitt et al., 2014). Feng et al. (2019) found a direct positive effect of perceived prejudice on psychological distress among Chinese male nursing students, but this effect was not strong; also, those male nursing students with high self-esteem tended to perceive less prejudice and thus report lower psychological distress. Self-esteem is a practical resource for coping with stress (Taylor and Stanton, 2007). Studies have shown that self-esteem contributes significantly to nursing students’ stress coping, influences stress coping levels (Yildirim et al., 2017), and is associated with positive coping behaviors (Lo, 2002). Lazarus and Folkman’s coping theory holds that cognitive assessment plays a vital role in the occurrence and response to stress (Folkman et al., 1986). When social prejudice cannot be temporarily eliminated, male nursing students with high self-esteem may adopt more positive cognitive appraisals and thus perceive less prejudice, thereby buffering stress. Therefore, we explored whether self-esteem indirectly affects professional identity by influencing perceived prejudice and, thus, psychological distress. The aim is to provide more informative information on reducing perceived prejudice, reducing psychological distress, and improving professional identity among male nursing students and male nurses. In addition, few studies discuss the mediating mechanisms that combine perceived prejudice and psychological distress.

Therefore, the present study proposes hypothesis 4: perceived prejudice positively predicts psychological distress, and the effect of self-esteem on professional identity can arise indirectly through this chain path: Self-esteem → Perceived prejudice → Psychological distress → Professional identity.

## 2. Materials and methods

### 2.1. Design and participants

This study is a cross-sectional study. The survey subjects were male nursing students studying for a full-time undergraduate degree or higher education and male nurses already working. The sample participating in this study consisted of 296 male nursing students and 168 male nurses aged 17–52 (*M* = 22.52 years, SD = 4.35 years). Additional sociodemographic characteristics of the sample are shown in Table 1.

**Table 1:** Self-esteem, perceived prejudice, psychological distress, and professional identity according to participant characteristics (*N* = 464).

Characteristic	*n* (%)	Prevalence of psychological distress (≥16) [*n* (%)]	*M* ± SD			
			Self-esteem	Perceived prejudice	Psychological distress	Professional identity
Age (years)						
<20	162 (34.9)		29.136 ± 4.774	2.154 ± 0.595	21.889 ± 7.350	58.253 ± 10.444
20–25	243 (52.4)		29.280 ± 4.611	2.191 ± 0.602	22.910 ± 7.829	56.626 ± 11.603
25–30	42 (9.1)		29.262 ± 4.829	2.282 ± 0.564	23.262 ± 7.497	54.238 ± 11.501
>30	17 (3.7)		31.706 ± 5.253	2.422 ± 0.527	22.824 ± 7.204	57.882 ± 10.265
*F*			1.544	1.381	0.715	1.665
Educational level						
Junior college or below	89 (19.2)		29.157 ± 4.753	2.006 ± 0.571	22.090 ± 8.458	59.337 ± 11.143
Bachelor	354 (76.3)		29.223 ± 4.637	2.241 ± 0.584	22.780 ± 7.412	56.531 ± 10.968
Master or above	21 (4.5)		31.571 ± 5.591	2.222 ± 0.727	21.333 ± 7.186	55.524 ± 13.920
*F*			2.533	5.690^**^	0.588	2.453
Male nursing students or Male nurses						
Male nursing students	296 (63.8)	241 (81.4)	29.557 ± 4.654	2.167 ± 0.593	22.047 ± 7.085	57.835 ± 11.162
Male nurses	168 (36.2)	138 (82.1)	28.893 ± 4.819	2.244 ± 0.596	23.524 ± 8.387	55.595 ± 11.101
*t*			1.459	−1.348	−1.925	2.081^*^
*χ^2^*		0.038				
First-choice major						
Nursing	289 (62.3)	226 (78.2)	29.595 ± 4.715	2.094 ± 0.575	21.886 ± 7.868	58.910 ± 11.142
Non-nursing	175 (37.7)	153 (87.4)	28.857 ± 4.705	2.361 ± 0.590	23.731 ± 7.026	53.909 ± 10.557
*t*			1.635	−4.797^***^	−2.548^*^	4.779^***^
*χ^2^*		6.203^*^				
Total	464 (100)	379 (81.7)	29.317 ± 4.720	2.195 ± 0.595	22.582 ± 7.607	57.024 ± 11.180

*M*, mean; SD, standard deviation; *p<0.05; **p<0.01; ***p<0.001.

### 2.2. Data collection

From November 2021 to January 2022, purposive and snowball sampling surveys were conducted in China. A total of 492 online electronic and paper questionnaires were distributed and returned, of which 464 were valid (an effective rate of 94.309%). All participants gave informed consent to this study and completed the questionnaires anonymously (without their names and the names of their workplaces).

### 2.3. Instrument

#### 2.3.1. The basic information questionnaire

We made the Basic Information Questionnaire based on the needs of this study, including age, educational level, a male nursing student or a male nurse, and whether nursing was the first-choice major.

#### 2.3.2. The male nursing students’ perceived prejudice questionnaire

The Male Nursing Students’ Perceived Prejudice Questionnaire was developed and made public in an English version by Feng et al. (2019) to measure the perceived prejudice of male nursing students in China. The questionnaire has a total of 6 items, and each item is scored on a four-point scale ranging from 1 (strongly disagree) to 4 (strongly agree). The average score for all items was calculated, with a higher score indicating stronger perceived prejudice. All the items on this questionnaire are also suitable for male nurses, so we contacted Feng by e-mail and used the Chinese version of the questionnaire she provided to measure the perceived prejudice of male nurses and male nursing students. In the study, Cronbach’s alpha coefficient for the questionnaire was 0.860.

#### 2.3.3. The 10-item Kessler psychological distress scale

The 10-item Kessler Psychological Distress Scale (K10) (Kessler et al., 2002) has a total of 10 items, and each item is scored on a five-point scale ranging from 1 (none of the time) to 5 (all of the time). The scores of the 10 items were summed up to obtain the total score, with a higher score indicating more serious psychological distress. A total score of 16 or above indicates very high psychological distress (Lyons et al., 2020). The Chinese version of K10 has also been shown to have good reliability and validity (Zhou et al., 2008). In the study, Cronbach’s alpha coefficient for the scale was 0.937.

#### 2.3.4. The professional identity questionnaire of nursing students

The Professional Identity Questionnaire of Nursing Students (PIQNS) was developed by Hao (2011) to measure the professional identity of nursing students in China. The questionnaire includes five factors: professional self-concept; benefits of staying and risks of leaving; social comparison and self-reflection; the autonomy of career choice; and social persuasion. We used it to measure male nursing students’ and male nurses’ professional identities (Chen et al., 2020). The questionnaire has a total of 17 items, and each item is scored on a five-point scale ranging from 1 (strongly disagree) to 5 (strongly agree). The scores of the 17 items were summed up to obtain the total score, with a higher score indicating a higher level of professional identity. In the study, Cronbach’s alpha coefficient for the questionnaire was 0.926.

#### 2.3.5. The Chinese version of the Rosenberg self-esteem scale

Mengcheng Wang et al. revised the Rosenberg Self-Esteem Scale (RSE) (Rosenberg, 1965) in Chinese to measure an individual’s level of self-esteem (Dai, 2010). The scale has a total of 10 items, and each item is scored on a four-point scale ranging from 1 (strongly disagree) to 4 (strongly agree). The scores of the 10 items were summed up to obtain the total score, with a higher score indicating a higher level of self-esteem. In the study, Cronbach’s alpha coefficient for the scale was 0.860.

#### 2.3.6. Data analysis

SPSS 25.0 was used for common method biases test, descriptive statistics, t-test, one-way ANOVA, chi-square test, and Pearson correlation analysis. The mediating effect was analyzed using model 6 in the PROCESS macro 3.3 developed by Hayes (2013), and the bias-corrected percentile bootstrap method was used to test the significance of the mediation effect.

## 3. Results

### 3.1. Common method biases test

Harman’s single-factor test was used to test for common method bias, and exploratory factor analysis was conducted on all measures’ items. The results showed that there were 6 factors with eigenvalues greater than 1, and the first factor explained 31.237% of the variation (less than 40%), which indicated that the study had no significant common method bias (Podsakoff et al., 2003).

### 3.2. Self-esteem, perceived prejudice, psychological distress, and professional identity according to participant characteristics

One-way ANOVA was used to first test the scores of each variable among different age groups and then to test the scores of each variable among different educational levels. The results showed that there was no statistically significant difference in the scores of each variable among different age groups, but there were statistically significant differences in the scores of perceived prejudice among different educational levels (*F* = 5.690, *p* < 0.01). Further post-hoc multiple comparisons showed that male nurses and male nursing students with an educational level of bachelor’s degree had significantly higher perceived prejudice scores than those with an educational level of junior college or below (*p* < 0.01). Then, the scores of each variable were compared between the male nurses and the male nursing students and between the participants who applied for the nursing major as their first choice and those who did not, using independent sample *t*-tests. The results showed that male nursing students scored significantly higher on professional identity than male nurses (*t* = 2.081, *p* < 0.05). Participants who applied for the nursing major as their first choice had significantly lower perceived prejudice scores and psychological distress scores than those who did not apply (*t* = −4.797, *p* < 0.001; *t* = −2.548, *p* < 0.05). Those who did apply also had significantly higher scores for professional identity than those who did not (*t* = 4.779, *p* < 0.001). In addition, the overall prevalence of psychological distress among male nurses and male nursing students was 81.7%. Using the chi-square test, we found no statistically significant difference in the prevalence of psychological distress between the male nurses and the male nursing students. Then the chi-square test was used again to compare the prevalence of psychological distress between the participants who applied for nursing as a first-choice major and those who did not. The results showed that the prevalence of psychological distress was significantly higher in the latter than in the former (*χ^2^* = 6.203, *p* < 0.05; See Table 1).

### 3.3. Correlation analysis of all variables

The results showed that self-esteem was significantly negatively correlated with perceived prejudice and psychological distress; both perceived prejudice and psychological distress were significantly negatively correlated with professional identity; perceived prejudice was significantly positively correlated with psychological distress; self-esteem was significantly positively correlated with professional identity. (See Table 2).

**Table 2:** Correlation matrix of all variables (*N* = 464).

Variables	1	2	3	4
1. Self-esteem	1			
2.Perceived prejudice	−0.244^**^	1		
3.Psychological distress	−0.504^**^	0.232^**^	1	
4. Professional identity	0.462^**^	−0.413^**^	−0.405^**^	1

**p< 0.01.

### 3.4. The mediating effects of perceived prejudice and psychological distress

**Figure 1:**
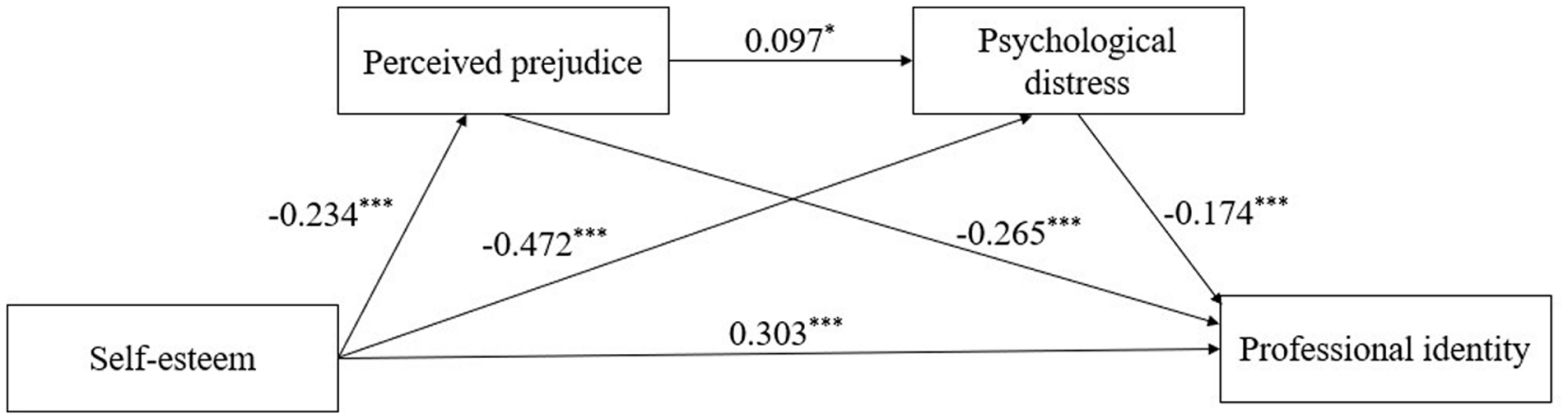
The multiple mediation model and each path coefficient. *p < 0.05; ***p < 0.001.

Model 6 in PROCESS was selected to test the mediating effects of the multiple mediation model, with psychological distress and perceived prejudice as the mediating variables, professional identity as the dependent variable, self-esteem as the independent variable, and controlling for the effects of educational level, male nursing students or male nurses, and first-choice major. The multiple mediation model of this study involves the mediation of perceived prejudice, the mediation of psychological distress, and the chain mediation of perceived prejudice and psychological distress (see Figure 1). The results of regression analysis among the variables were as follows (see Table 3): self-esteem significantly positively predicted professional identity (*β* = 0.451, *p* < 0.001); after incorporating self-esteem, perceived prejudice, and psychological distress into the regression equation at the same time, the predictive effect of self-esteem on professional identity was still significant (*β* = 0.303, *p* < 0.001); self-esteem could significantly negatively predict perceived prejudice (*β* = −0.234, *p* < 0.001) and psychological distress (*β* = −0.472, *p* < 0.001); perceived prejudice significantly positively predicted psychological distress (*β* = 0.097, *p* < 0.05); and perceived prejudice and psychological distress significantly negatively predicted professional identity, respectively (*β* = −0.265, *p* < 0.001; *β* = −0.174, *p* < 0.001).

**Table 3:** Regression analysis between variables (*N* = 464).

Regression equation		Overall fitness index			Significance of regression coefficient	
Outcome variable	Predictive variable	*R*	*R^2^*	*F*	*β*	*t*
Professional identity		0.512	0.262	40.791		
	Educational level				−0.126	−2.867^**^
	Male nursing students or Male nurses				−0.096	−2.215^*^
	First-choice major				−0.147	−3.502^***^
	Self-esteem				0.451	11.195^***^
Perceived prejudice		0.342	0.117	15.201		
	Educational level				0.141	2.947^**^
	Male nursing students or Male nurses				0.081	1.709
	First-choice major				0.162	3.532^***^
	Self-esteem				−0.234	−5.315^***^
Psychological distress		0.523	0.274	34.544		
	Educational level				0.038	0.858
	Male nursing students or Male nurses				0.064	1.481
	First-choice major				0.046	1.106
	Self-esteem				−0.472	−11.503^***^
	Perceived prejudice				0.097	2.285^*^
Professional identity		0.595	0.354	41.689		
	Educational level				−0.080	−1.913
	Male nursing students or Male nurses				−0.062	−1.521
	First-choice major				−0.093	−2.336^*^
	Self-esteem				0.303	6.857^***^
	Perceived prejudice				−0.265	−6.564^***^
	Psychological distress				−0.174	−3.924^***^

*p<0.05; **p<0.01; ***p<0.001.

The direct effect, specific mediating effect, comparative mediating effect, and total mediating effect were tested for significance using the bias-corrected nonparametric percentile bootstrap method (95% confidence intervals were estimated after 5,000 repeated sampling) (Fang et al., 2014). These effects are significant if the 95% confidence intervals do not contain 0. The results of the mediating effect analysis were as follows (see Table 4): the effect value of indirect path 1 (indirect1) consisting of Self-Esteem → Perceived Prejudice → Professional Identity was 0.062 [95% CI (0.031, 0.099)]; the effect value of indirect path 2 (indirect2) consisting of Self-Esteem → Psychological Distress → Professional Identity was 0.082 [95% CI (0.033, 0.137)]; the effect value of indirect path 3 (indirect3) consisting of Self-Esteem → Perceived Prejudice → Psychological Distress → Professional Identity was 0.004 [95% CI (0.000, 0.010)]; the total mediating effect value (indirect + indirect2 + indirect3) was 0.148 [95% CI (0.090, 0.211)]; and the effect value of direct path (direct) consisting of Self-Esteem → Professional Identity was 0.303 [95% CI (0.20, 0.401)]. All the above Bootstrap 95% confidence intervals do not contain 0, indicating that the chain mediating effect of perceived prejudice and psychological distress, the mediating effect of psychological distress, the mediating effect of perceived prejudice, and the direct effect of self-esteem on professional identity were all significant. The total mediating effect accounted for 32.816% of the total effect, and the direct effect accounted for 67.184% of the total effect.

**Table 4:** Decomposition of effects in the impact of self-esteem on professional identity (*N* = 464).

Effect	Path	Effect value	SE	95% Confidence Interval		Effect ratio
				Lower	Upper	
Mediation effect	Indirect1	0.062	0.018	0.031	0.099	13.747%
	Indirect2	0.082	0.026	0.033	0.137	18.182%
	Indirect3	0.004	0.003	0.000	0.010	0.887%
Total mediation effect	Indirect1 + indirect2 + indirect3	0.148	0.031	0.090	0.211	32.816%
Direct effect	Direct	0.303	0.051	0.201	0.401	67.184%
Total effect	Indirect1 + indirect2 + indirect3 + direct	0.451	0.045	0.364	0.540	100.000%
Indirect effect contrast	Indirect1-indirect2	−0.020	0.034	−0.088	0.046	
	Indirect1-indirect3	0.058	0.017	0.028	0.094	
	Indirect2-indirect3	0.078	0.025	0.031	0.130	

Indirect1: Self-Esteem → Perceived Prejudice → Professional Identity; indirect2: Self-Esteem → Psychological Distress → Professional Identity; indirect3: Self-esteem → Perceived Prejudice → Psychological Distress → Professional Identity; direct: Self-Esteem → Professional Identity.

## 4. Discussion

This study explored the impact of male nurses’ and male nursing students’ self-esteem on their professional identity and its internal mechanisms. The results showed that male nurses’ and male nursing students’ self-esteem could not only directly affect their professional identity but also indirectly affect it through the mediating effect of perceived prejudice, the mediating effect of psychological distress, and the chain mediating effect of perceived prejudice and psychological distress. This study designed and verified this multiple mediation model based on social identity theory and coping theory (Tajfel and Turner, 1979; Folkman et al., 1986). It aims to emphasize the relationship between the self-esteem and professional identity of male nursing students and male nurses in a particular environment where stereotypes and social prejudice exist and to provide diverse reference information on how to improve the professional identity of male nursing students and male nurses. Stereotypes and societal prejudice against male nursing students and nurses should be seen, acknowledged, and valued to promote an equitable, gender-diverse environment.

In addition, this study found that male nurses and male nursing students with an educational level of bachelor’s degree had higher perceived prejudice than those with an educational level of junior college or below. The status of nurses is relatively low-slung in China, and the public perception is that nurses are poorly educated, subordinate to doctors, and do work that is not very technical (Ma, 2019). Even though nurses with an educational level of bachelor’s degree are better educated than those with an educational level of junior college or below, there is no difference in the tasks they perform in the hospital. As a result, it may lead to stronger social prejudice being perceived by those male nurses and male nursing students who with an educational level of bachelor’s degree. A previous study has found that male nursing students at three-year colleges had a higher professional identity than junior male nurses (Chen et al., 2020); consistent with it, this study found that male nursing students had a higher professional identity than male nurses. However, the research subjects included in this study are more representative. In China, full-time undergraduate nursing students, that is, four-year nursing students, have become mainstream. In addition, the research objects of this study also include male nursing students with higher education and male nurses with unlimited seniority. From this point of view, the research objects of this study are more diverse and more representative, which is also one of the innovations of this study. Compared to male nursing students, male nurses may experience more and more stress in the workplace, leading to a decreased sense of professional identity (Chen et al., 2022). Therefore, nursing managers should take steps to reduce the stress of male nurses, which includes, but is not limited to, perceived prejudice and may include other work-related stressors. Comparing male nurses and male nursing students who applied for the nursing major as their first choice with those male nurses and male nursing students who did not apply, the following findings were found: the former felt less social prejudice and had a lower prevalence of psychological distress, which was consistent with the previous findings on male nursing students (Feng et al., 2019); the former had a higher professional identity, which matched the earlier discoveries (Zhang et al., 2018; Zhou et al., 2021); and the former had lower psychological distress, which was inconsistent with previous studies on male nursing students that found no statistically significant difference between the two in terms of psychological distress (Feng et al., 2019). The inconsistency with the previous finding may be because the subjects of this study were recruited on a larger scale rather than being limited to a particular province in China. From this point of view, nursing educators and managers cannot ignore the care, support, and guidance for male nursing students and male nurses who did not first choose a nursing major to avoid losing them. Because of the current admission system and shortage of nurses in China, nursing schools will continue to admit male students who are assigned to the nursing major because their grades do not meet the requirements of other majors. Therefore, the attitudes and practices of nursing educators and managers toward this population of male nursing students and male nurses are critical.

### 4.1. Direct impact

This study showed that the self-esteem of male nursing students and male nurses significantly positively predicted their professional identity, consistent with previous findings on other populations (Motallebzadeh and Kazemi, 2018; Min et al., 2021), and the research H1 was verified. Notably, the direct effect of self-esteem on professional identity among male nursing students and male nurses accounted for 67.184% of the total effect, indicating that the direct effect of self-esteem on professional identity is essential. Therefore, nursing educators and administrators should consistently strive to protect the self-esteem of male nurses and male nursing students and improve their self-esteem. For example, (a) emphasize the importance of male nursing students and male nurses to nursing and provide them with timely and positive feedback when they achieve success; (b) invite men who have been successful in their nursing careers to share and exchange experiences with male nursing students and male nurses so that role models can also enhance the self-esteem of male nursing students and male nurses.

### 4.2. Mediating role of perceived prejudice

This study showed that the self-esteem of male nurses and male nursing students affected their professional identity by affecting perceived prejudice, and the research H2 was verified. The persistence of stereotypes and social prejudice against male nursing students and male nurses is a cause for concern. Self-esteem and professional identity are important for male nursing students and male nurses, and further exploration of the relationship between the two in an environment where stereotypes and social prejudice exist is necessary. However, there is a paucity of research on male nursing students’ and male nurses’ self-esteem and professional identity, and in particular, no studies have yet taken the effects of social prejudice into account. Therefore, we explored the mediating role of perceived prejudice among male nursing students and male nurses in the relationship between self-esteem and professional identity. Male nurses’ and male nursing students’ self-esteem could significantly negatively predict their perceived prejudice, which was in line with previous findings on male nursing students (Feng et al., 2019) and other populations (Ji et al., 2019; Maqsood et al., 2021). Perceived prejudice among male nursing students and male nurses significantly and negatively predicted professional identity, which is consistent with the findings of a previous qualitative study in which some male nurses said they had experienced social prejudice from their nursing student days to their working years and that it negatively affected their professional identity (Zhang, 2019). This study examined how male nursing students’ and male nurses’ perceived prejudice affects their professional identity, filling a gap in quantitative research on this issue. Perceived prejudice among male nursing students and male nurses is a stressor. In the presence of continued societal prejudice against male nursing students and male nurses, high self-esteem may motivate them to use positive cognitive appraisals, which allow them to perceive less prejudice and thus have a higher professional identity. The higher the professional identity, the more they may want to stay in nursing (Johnson et al., 2012; Zhang et al., 2017). Therefore, nursing educators and administrators can reduce the perceived prejudice of male nursing students and male nurses by protecting and improving their self-esteem, thereby improving their professional identity. Actually, one of the reasons men pursue a nursing career is out of helpfulness (Stanley et al., 2016). In addition, male nurses possess some advantages, such as good physical strength, usually calm decision-making in case of emergencies, and an excellent ability to operate medical equipment, which makes them more adapted to work in the intensive care unit, emergency department, psychiatric department, and operating room (Zhang and Tu, 2020). The general public should recognize male nursing students and male nurses, and it is necessary to reduce the perceived prejudice of male nursing students and male nurses. Reducing the perceived prejudice of male nursing students and male nurses should be done in two ways: on the one hand, their self-esteem level should be improved; on the other hand, social awareness and understanding of male nursing students and male nurses can be popularized through the media, thus reducing the prejudice against them and recognizing their profession and work.

### 4.3. Mediating role of psychological distress

This study showed that the self-esteem of male nurses and male nursing students affected their professional identity by affecting psychological distress, and the research H3 was verified. First, their self-esteem could significantly negatively predict their psychological distress, which was in line with previous findings on male nursing students (Feng et al., 2019) and nurses (Feng et al., 2018; Duran et al., 2021; Liu et al., 2021). Thus, the role of self-esteem in promoting mental health was reaffirmed, which supports the previous views (Mann et al., 2004). Then, their psychological distress significantly negatively predicted their professional identity, similar to previous studies that found that college students’ levels of psychological well-being positively predicted professional identity (Strauser et al., 2008). However, there is a dearth of research on the role of mental health in career development (Strauser et al., 2008), particularly in the male nursing student and male nurse populations. Thus, the present study contributes to this. Overall, male nursing students and male nurses with higher levels of self-esteem are likely to experience lower levels of psychological distress and may have a higher sense of professional identity. By examining psychological distress as a mediating variable between self-esteem and professional identity, this study not only focused on the psychological health of male nursing students and male nurses but also linked their psychological distress to their self-esteem and professional identity. The study’s results revealed that nursing educators and administrators could reduce the psychological distress of male nursing students and male nurses by protecting and enhancing their self-esteem, thereby enhancing their professional identity. In recent years, psychological distress among nurses has received increasing attention (Chiu, 2022). However, in China, the number of male nursing students and male nurses is too small relative to their female counterparts. So even in studies on psychological distress among nursing students and nurses (Liu et al., 2021; Qi et al., 2022), the findings may be more in line with the situation of female nursing students and female nurses. Thus, this study was conducted on male nursing students and male nurses to present their psychological distress situations more clearly. Among the male nurses and male nursing students who took part in this study, psychological distress was present in as many as 81.7% of them, which was similar to the results of a previous study on male nursing students (Feng et al., 2019). Not only does this indicate that their physical and mental health is at risk, but it has the potential to reduce their sense of professional identity. In general, it is important to pay attention to the mental health of male nurses and male nursing students and take measures to alleviate their psychological distress. Hence, hospitals and schools can hold more lectures on mental health, screen for the prevalence of psychological distress promptly, and provide psychological assistance to male nurses and male nursing students when necessary. Also, it is very crucial to protect and improve their self-esteem to ease psychological distress.

### 4.4. The chain mediating effect of perceived prejudice and psychological distress

This study also found that the self-esteem of male nurses and male nursing students could indirectly affect professional identity through the chain mediating effect of perceived prejudice and psychological distress, and research H4 was verified. The prejudice perceived by male nurses and male nursing students could impair their mental health and positively predict psychological distress among them, which was in line with what has been found about male nursing students (Feng et al., 2019) and other groups (Schmitt et al., 2014; Feng and Xu, 2015; Liu and Zhao, 2016; Drabish and Theeke, 2022). However, the effect of perceived prejudice on psychological distress was relatively small, which was consistent with previous research conclusions on male nursing students (Feng et al., 2019). Male nurses and male nursing students may also suffer from psychological distress due to other stressors, but those with positive coping styles, good social support, and good psychological resilience may be able to resist psychological distress (Watson et al., 2009; Klainin-Yobas et al., 2014; Smith and Yang, 2017; Feng et al., 2018; Foster et al., 2020). Therefore, future studies can explore factors besides self-esteem that can reduce psychological distress among male nursing students and male nurses, thus improving professional identity.

Male nursing students and male nurses are in an environment where stereotypes and social prejudice exist, and for them, psychological distress caused by perceived prejudice may be a particular experience. Therefore, the impact of their perceived prejudice on psychological distress cannot be ignored, and this study explored the chain mediation role of perceived prejudice and psychological distress in the relationship between self-esteem and professional identity. The chain-mediating effect of perceived prejudice and psychological distress accounted for only 0.887% of the total effect. The chain-mediating effect was weak and significantly smaller than the independent mediating effects of perceived prejudice and psychological distress. Although it was weak, the effect value was still statistically significant. We cannot ignore the chain mediating effect of perceived prejudice and psychological distress. Social prejudices against male nursing students and male nurses still exist and are difficult to eliminate. In this context, as mentioned in coping theory (Folkman et al., 1986), cognitive appraisal plays a critical role in the occurrence and response to stress. Therefore, male nursing students and male nurses with high self-esteem may adopt more positive cognitive appraisals and thus have lower perceived prejudice, thereby reducing stress. As a result, they may tend to suffer from lower psychological distress and have a higher professional identity. Therefore, to improve the professional identity of male nursing students and male nurses, we can still start from this path: improve self-esteem → reduce perceived prejudice → reduce psychological distress → improve professional identity.

### 4.5. Limitations

First, this study was cross-sectional, which could not prove the causal relationship between variables; longitudinal research or experimental research can be used to determine the causal relationship between variables. Second, the participants in this study were all from China. Due to differences in culture, education, and management, it may not be possible to generalize all results to other countries; future research should examine the significance of other samples in this model. Third, this study did not examine the differences between male nursing students in different grades and male nurses in different departments on each variable; future studies could improve on this. Fourth, because of the difficulty of obtaining a sample and the fact that male nursing students and male nurses are essentially a common group, this study put male nursing students and male nurses together for research; in the future, they can be separated for more targeted research to provide more targeted and specific reference information for nursing educators or nursing managers.

## 5. Conclusion

(1) Male nurses’ and male nursing students’ self-esteem could directly and positively affect their professional identity, and this direct effect cannot be underestimated.
(2) Male nurses’ and male nursing students’ self-esteem could indirectly affect their professional identity through the mediating role of perceived prejudice, the mediating role of psychological distress, and the chain-mediating role of perceived prejudice and psychological distress.
(3) Male nurses and male nursing students had a high prevalence of psychological distress, and their mental health needed attention.
(4) In general, improving the professional identity of male nursing students and male nurses can start with the following aspects: protecting and improving their self-esteem; reducing prejudice against them; valuing their mental health and alleviating their psychological distress.

## Data availability statement

The raw data supporting the conclusions of this article will be made available by the authors, without undue reservation.

## Ethics statement

The studies involving human participants were reviewed and approved by the study was approved by the Human Research Ethics committee of Kunming Medical University (ethical approval number: 2022kmykdx6f68). Written informed consent to participate in this study was provided by the participants’ legal guardian/next of kin.

## Author contributions

XW, WW, and YZ: conception and design. XW, JP, JL, XY, QL, YZ, XZ, ZG, XC, and FT: data collection. XW and XM: analysis and interpretation of data. XW: writing the manuscript. XW, XY, JP, and JL: critical revision of the manuscript. WW: statistical expertise. XY, YZ, and YZ: obtaining funding. YZ: administrative, technical, or material support. YZ: supervision. All authors have contributed significantly to this study and all authors are in agreement with the manuscript.

## Funding

This study was supported by National Natural Science Foundation of China, grant no. 81960254, 82060257, and 82260276; Joint special fund of Applied Fundamental Research of Kunming Medical University granted by Science and Technology Office of Yunnan, grant no. 202101AY070001-196 and 202201AY070001-181.

## Conflict of interest

The authors declare that the research was conducted in the absence of any commercial or financial relationships that could be construed as a potential conflict of interest.

## Publisher’s note

All claims expressed in this article are solely those of the authors and do not necessarily represent those of their affiliated organizations, or those of the publisher, the editors and the reviewers. Any product that may be evaluated in this article, or claim that may be made by its manufacturer, is not guaranteed or endorsed by the publisher.
