# Supplementary material for: Self-esteem and professional identity among male nurses and male nursing students: mediating roles of perceived prejudice and psychological distress
Source: Front Psychol. 2023 Jun 13;14:1176970. doi: 10.3389/fpsyg.2023.1176970 (PMC10294685; doi:10.3389/fpsyg.2023.1176970)
Supplement: Supplementary file 1 [file Data_Sheet_1.docx]

**A questionnaire on male nursing students and male nurses**

Dear nursing colleagues, hello!

I am a master's student in applied psychology from Kunming Medical University. Like you, I was once a nursing staff, and my four years of undergraduate study and one year of work experience have given me a deeper understanding and feeling of the nursing profession. Although more and more men are now joining the nursing profession, the number of men still needs to catch up to the number of women, and the care and attention men receive still need to be increased. I plan to use this research study to find out whether you perceive social prejudice and to focus on your psychological distress, professional identity, and self-esteem. I hope to provide some reference information for nursing education and management and do my part to help society better understand male nursing students.

Therefore, please spare some precious time to answer the following questionnaire truthfully. I will survey anonymously, your participation is precious to this study, and the survey data will be used for scientific research only. The survey data will be confidential, so please feel free to fill it out. This questionnaire is divided into five sections with a total of 48 questions. Please read the instructions at the beginning of each section carefully and then answer the questionnaire carefully. Thank you for your cooperation!

The Psychiatric and Psychological Subject Group of the Second Affiliated Hospital of Kunming Medical University

**Part I: This is the basic information; please fill in your personal situation truthfully, and tick a box below questions that best represents how you have been.**

1. Your age (years): ( )

2. Your current level of education:

□Junior college or below □Bachelor □Master or above

3. Which of the following stages are you currently in:

□Undergraduate student □Master's student □Doctoral student □Already working

4. Which province in China are you currently studying or doing an internship or working in: ( )

5. Your first-choice major at one time was:

□Nursing □Other major not in nursing

**Part II: These questions were designed to find out if you, as male nursing students or male nurses, feel any social prejudice against you.** **Tick a box below each question that best represents how you have been.** **There are no good, bad, correct, or wrong answers; your true feelings are of deep value to this study. Thank you for your cooperation!**

1. The public perception is that only women are suitable to be nurses.

□Strongly disagree □Disagree □Agree □Strongly agree

2. The public perception is that men are not suitable to be nurses.

□Strongly disagree □Disagree □Agree □Strongly agree

3. Most people around me believe that women are more suitable to study nursing than men.

□Strongly disagree □Disagree □Agree □Strongly agree

4. Most people around me believe that boys are not suitable to study nursing.

□Strongly disagree □Disagree □Agree □Strongly agree

5. When I interact with people, I do not want them to know that I am majoring in nursing.

□Strongly disagree □Disagree □Agree □Strongly agree

6. My major is an obstacle to my love and marriage.

□Strongly disagree □Disagree □Agree □Strongly agree

**Part III: These questions concern how you have been feeling over the past 30 days. Tick a box below each question that best represents how you have been.** **There are no good, bad, correct, or wrong answers; your true feelings are of deep value to this study. Thank you for your cooperation!**

1. During the last 30 days, about how often did you feel tired out for no good reason?

□None of the time □A little of the time □Some of the time □Most of the time □All of the time

2. During the last 30 days, about how often did you feel nervous?

□None of the time □A little of the time □Some of the time □Most of the time □All of the time

3. During the last 30 days, about how often did you feel so nervous that nothing could calm you down?

□None of the time □A little of the time □Some of the time □Most of the time □All of the time

4. During the last 30 days, about how often did you feel hopeless?

□None of the time □A little of the time □Some of the time □Most of the time □All of the time

5. During the last 30 days, about how often did you feel restless or fidgety?

□None of the time □A little of the time □Some of the time □Most of the time □All of the time

6. During the last 30 days, about how often did you feel so restless you could not sit still?

□None of the time □A little of the time □Some of the time □Most of the time □All of the time

7. During the last 30 days, about how often did you feel depressed?

□None of the time □A little of the time □Some of the time □Most of the time □All of the time

8. During the last 30 days, about how often did you feel that everything was an effort?

□None of the time □A little of the time □Some of the time □Most of the time □All of the time

9. During the last 30 days, about how often did you feel so sad that nothing cheer you up?

□None of the time □A little of the time □Some of the time □Most of the time □All of the time

10. During the last 30 days, about how often did you feel worthless?

□None of the time □A little of the time □Some of the time □Most of the time □All of the time

**Part IV: These questions aimed to understand the career development of male nursing students and male nurses.** **Tick a box below each question that best represents how you have been. There are no good, bad, correct, or wrong answers; your true feelings are of deep value to this study. Thank you for your cooperation!**

1. I would love to be a nurse.

□Strongly disagree □Somewhat disagree □Intermediate □Somewhat agree □Strongly agree

2. I love learning about the growth stories of people who have succeeded in nursing.

□Strongly disagree □Somewhat disagree □Intermediate □Somewhat agree □Strongly agree

3. I want to talk to seniors in the nursing field.

□Strongly disagree □Somewhat disagree □Intermediate □Somewhat agree □Strongly agree

4. No matter what people say, I will choose the work I like.

□Strongly disagree □Somewhat disagree □Intermediate □Somewhat agree □Strongly agree

5. I have invested too much in my nursing career (e.g., education and personal effort) and would not like to change careers now.

□Strongly disagree □Somewhat disagree □Intermediate □Somewhat agree □Strongly agree

6. I will not change the direction of my current career.

□Strongly disagree □Somewhat disagree □Intermediate □Somewhat agree □Strongly agree

7. I often reflect on my interests, personality, abilities, and values to explore my future career development.

□Strongly disagree □Somewhat disagree □Intermediate □Somewhat agree □Strongly agree

8. Nursing allows me to develop my abilities and strengths.

□Strongly disagree □Somewhat disagree □Intermediate □Somewhat agree □Strongly agree

9. I like my major and am ready to actively develop in this direction.

□Strongly disagree □Somewhat disagree □Intermediate □Somewhat agree □Strongly agree

10. Leaving the nursing profession would cause me some emotional trauma.

□Strongly disagree □Somewhat disagree □Intermediate □Somewhat agree □Strongly agree

11. I am proud to be in the nursing field.

□Strongly disagree □Somewhat disagree □Intermediate □Somewhat agree □Strongly agree

12. My knowledge of careers came entirely from the indoctrination of my parents, teachers, or other authorities, without any serious consideration on my part.

□Strongly disagree □Somewhat disagree □Intermediate □Somewhat agree □Strongly agree

13. I have tried understanding the situation in various fields to strengthen my professional beliefs.

□Strongly disagree □Somewhat disagree □Intermediate □Somewhat agree □Strongly agree

14. Nursing allows me to use my creativity.

□Strongly disagree □Somewhat disagree □Intermediate □Somewhat agree □Strongly agree

15. When choosing a job, in addition to considering your ideals, it is necessary to consider the influence of the external environment.

□Strongly disagree □Somewhat disagree □Intermediate □Somewhat agree □Strongly agree

16. I am happy to work as a nurse.

□Strongly disagree □Somewhat disagree □Intermediate □Somewhat agree □Strongly agree

17. I am sure that I can succeed in a nursing career.

□Strongly disagree □Somewhat disagree □Intermediate □Somewhat agree □Strongly agree

**Part V: These questions are designed to find out how you see yourself. Tick a box below each question that best represents how you have been. There are no good, bad, correct, or wrong answers; your true feelings are of deep value to this study. Thank you for your cooperation!**

1. On the whole, I am satisfied with myself.

□Strongly disagree □Disagree □Agree □Strongly agree

2. At times, I think I am no good at all.

□Strongly disagree □Disagree □Agree □Strongly agree

3. I feel that I have a number of good qualities.

□Strongly disagree □Disagree □Agree □Strongly agree

4. I am able to do things as well as most other people.

□Strongly disagree □Disagree □Agree □Strongly agree

5. I feel I do not have much to be proud of.

□Strongly disagree □Disagree □Agree □Strongly agree

6. I certainly feel uselessly at times.

□Strongly disagree □Disagree □Agree □Strongly agree

7. I feel that I’m a person of worth.

□Strongly disagree □Disagree □Agree □Strongly agree

8. I wish I could have more respect for myself.

□Strongly disagree □Disagree □Agree □Strongly agree

9. All in all, I am inclined to think that I am a failure.

□Strongly disagree □Disagree □Agree □Strongly agree

10. I take a positive attitude toward myself.

□Strongly disagree □Disagree □Agree □Strongly agree
